# Considerations on Immunization and Immunosuppression of Patients With Autoimmune Blistering Diseases During COVID-19 Pandemic in Brazil: Case Report

**DOI:** 10.3389/fmed.2021.811562

**Published:** 2022-04-12

**Authors:** Denise Miyamoto, Claudia Giuli Santi, Celina Wakisaka Maruta, Valeria Aoki

**Affiliations:** ^1^Department of Dermatology, Hospital das Clinicas HCFMUSP, Faculdade de Medicina, Universidade de São Paulo, São Paulo, Brazil; ^2^Department of Dermatology, Faculdade de Medicina FMUSP, Universidade de São Paulo, São Paulo, Brazil

**Keywords:** COVID-19, SARS-CoV-2, autoimmune blistering diseases, pemphigus, immunosuppressants, rituximab, vaccine

## Abstract

Autoimmune blistering diseases comprise a rare group of potentially life-threatening dermatoses. Management of autoimmune disorders poses a challenge in terms of achieving disease control and preventing adverse events. Treatment often requires an individualized approach considering disease severity, age, comorbidities, and infectious risk especially in the context of the ongoing COVID-19 pandemic. Knowledge regarding SARS-CoV-2 infection is still evolving and no specific antiviral therapy is available yet. We report four patients with active disease that required adjustment of treatment during the pandemic to discuss the use of immunosuppressants and immunobiologics, weighing potential risks and benefits of each therapy modality and vaccination status.

## Introduction

Since the COVID-19 outbreak, management of autoimmune blistering diseases (AIBD) became even more challenging to provide adequate immunosuppressive treatment while minimizing infectious risk. Clinicians recommend individualized approach considering disease severity, patients' age and comorbidities while no specific antiviral therapy is available.

Brazil has the third highest number of confirmed COVID-19 cases and the second highest mortality rate, with nearly 21.82 million cases and 608,000 deaths (1). The University of São Paulo Medical School is a reference center for AIBD, with 1,156 patients under follow-up (683 with pemphigus and 473 with subepidermal blistering diseases). From March until September 2020, our hospital exclusively dedicated 800 beds for the treatment of 4,500 severe COVID-19 cases (2), which limited dermatological outpatient and inpatient consultations (3). Such measures led to reschedule AIBD patients in remission and reevaluation of immunosuppressant therapy with the lowest immunosuppression for patients with active disease. We hereby report four cases that required treatment assessment during the pandemic to discuss the use of immunosuppressive and immunobiologics, weighing potential risks and benefits of each treatment and vaccination status (Table 1).

**Table 1:** Summary of AIBD cases treated during COVID-19 pandemic.

**No**.	**Age/sex**	**Diagnosis/** **duration**	**Comorbidities**	**Treatment** ^**a**^	**COVID-19 vaccine**	**COVID-19** **infection**^**b**^	**Outcome**
1	57/male	PF 3 months	Schizophrenia	Pred 80 mg/d (1 mg/kg/d) MMF 3 g/d	Unavailable	D57	Deceased
2	36/male	PV 8 months	Diabetes type I Obesity	Pred 15 mg/d (0.2 mg/kg/d) MMF 2 g/d RTX 1 g (Jan 18 and Feb 5, 2021)	Unavailable	D63	Recovery
3	45/female	PV 1 month	Pulmonary embolism	Pred 50 mg/d (0.7 mg/kg/d) RTX 1 g (Apr 22 and May 6, 2021)	Unavailable	D45	Deceased
4	61/male	PV 3 years	Diabetes type II	Pred 30 mg/d (0.4 mg/kg/d) RTX 1 g (Aug 30 and Sep 13, 2021)	PfizerBioNTech (May, Jul, Sep 2021)	N/A	N/A

a *Treatment in use at the time of COVID-19 infection*.

b *Interval between onset of immunosuppression and SARS-CoV-2 infection; MMF, mycophenolate mofetil; N/A, not applicable; PF, pemphigus foliaceus; Pred, prednisone; PV, pemphigus vulgaris; RTX, rituximab*.

## Case Description

### Patient 1

A 57-year-old male patient with schizophrenia presented diffuse blisters and confluent erosions on the face and trunk for 3 months. He was hospitalized on March 13, 2020, and the diagnosis of pemphigus foliaceus was confirmed: histopathological analysis revealed acantholysis and cleavage at the spinous layer level. Immunofluorescence findings showed intercellular intraepidermal deposits of IgG and C3 (direct immunofluorescence) and circulating IgG autoantibodies by indirect immunofluorescence (titers >1:2,560, intercellular epidermal pattern). Initial treatment started with oxacillin 1 g 4/4 h, methylprednisolone 80 mg/d and mycophenolate mofetil (MMF) 3 g/d. On D12, he developed multiple round crusts predominantly on the periocular region, diagnosed as Kaposi varicelliform eruption and received intravenous acyclovir 5 mg/kg/dose. Disease control was achieved on D35 and the patient was discharged with prednisone 80 mg/d and MMF 3 g/d. On April 27, 2020, during the first follow-up visit, the patient complained of weakness and fever (>100.4 F) for 1 day. Infectious disease clinicians recommended influenza vaccination and prescribed oseltamivir 75 mg BID for 5 days. Once the patient did not attend his 1 week-follow-up visit, we contacted his family, who informed that he was admitted in a different hospital and passed away due to COVID-19.

### Patient 2

**Figure 1:**
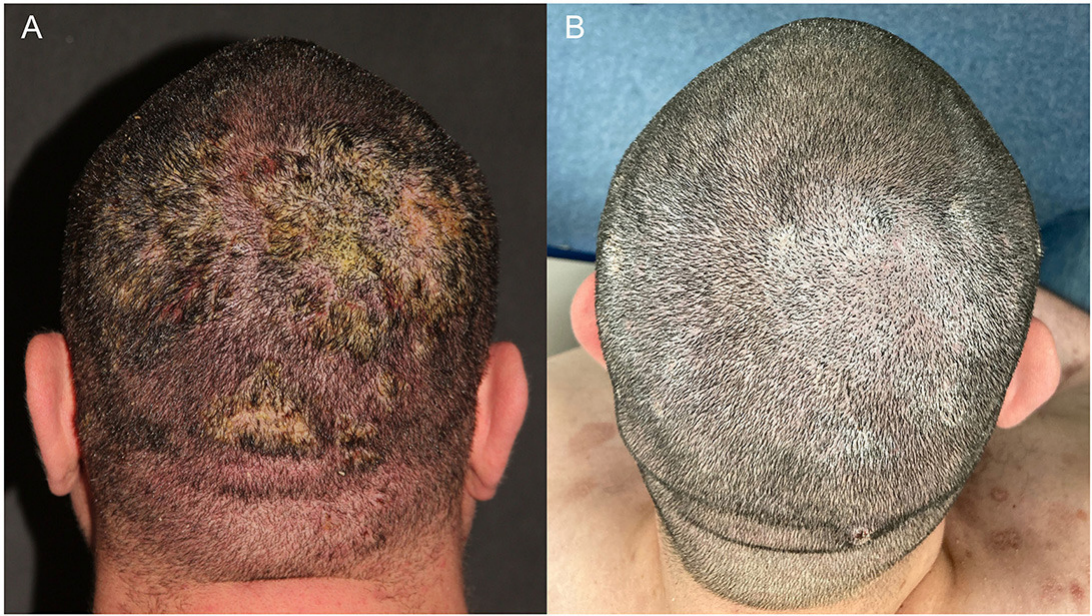
A 36-year-old man with pemphigus vulgaris. **(A)** Confluent erosions with purulent crusts on the scalp in February 2021. **(B)** Improvement of the lesions 9 months after rituximab treatment.

A 36-year-old male patient with refractory pemphigus vulgaris (PV) and uncontrolled type I diabetes was referred to our institution due to persistent erythematous and squamous plaques on the scalp and confluent erosions on the trunk for 8 months. His prior treatment included prednisone (40 mg/day), azathioprine (100 mg/day) and doxycycline (200 mg/day) since September 2020, prescribed elsewhere. In December 2020, we replaced azathioprine for MMF 3 g/day due to the refractoriness of PV lesions. Once the PV activity persisted and his comorbidities such as diabetes and obesity aggravated, we decided for rituximab (RTX), two 1 g infusions, administered on January 18 and February 5, 2021 (Figure 1A). Within 1 month after anti-CD20 therapy, the patient achieved partial remission, with complete healing of the PV lesions on the trunk, partial clearing of crusted plaques on the scalp and adequate control of the diabetes.

However, on Mar 31, 2021, he presented with fever, pustules and exsudative plaques on the scalp for 10 days despite treatment with prednisone 15 mg/day and mycophenolate mofetil 2 g/day. He was hospitalized and SARS-CoV-2 PCR was positive on D10. Thorax CT revealed multiple ground glass opacities with multifocal and bilateral areas of consolidation involving up to 50% of the lung parenchyma. He received Heparin 5,000 UI 12/12 h and oxacillin 1 g 4/4 h for the cutaneous infection., and PV treatment changed to monotherapy with prednisone 30 mg/d. On D13, he developed hypoxemia (O_2_ saturation = 88%) and required oxygen supplementation with nasal catheter (2 L/min) that progressed to non-invasive ventilation due to respiratory failure. On D16, he was transferred to the intensive care unit and put on awake prone ventilation; prednisone was replaced with dexamethasone 20 mg/day and heparin was increased to 5,000 UI 8/8 h. On D25 oxygen supplementation was progressively reduced and the patient was discharged after 27 days of hospitalization. He fully recovered of COVID-19 without sequelae. On October 25, 2021, during his last follow-up visit, PV was on remission with prednisone 7.5 mg/day (Figure 1B).

### Patient 3

A 45-year-old otherwise healthy female patient presented lesions on the scalp for 1 month that progressed to the trunk, abdomen, and limbs along with oral and vaginal erosions. On March 22, 2021, she was admitted to the hospital for diagnostic confirmation and treatment. Histopathological examination (abdomen) revealed a suprabasilar acantholytic dermatosis. Direct immunofluorescence demonstrated IgG, C3 and IgA intercellular deposits within the epidermis and IgM and C3 focally deposited at the basement membrane zone. Indirect immunofluorescence titers of IgG on human foreskin were of 1:640 and negative on transitional murine epithelium. We then confirmed the diagnosis of PV after a complete systemic workup with no evidence of neoplasia. Additional systemic findings revealed incidental acute bilateral pulmonary embolism without thrombophilia and no cardiac dysfunction that needed anticoagulation with rivaroxaban. After 30 days, the mucocutaneous PV erosions evolved with slow central healing. However, persistent PV activity occurred despite the use of prednisone 1.4 mg/kg/d and MMF 3 g/day, thus limiting the tapering of immunosuppression. She then received two infusions of RTX 1 g within 14 days. As the patient evolved with lymphopenia (600/mm^3^), MMF was withdrawn, and we added prophylactic trimethoprim/sulfamethoxazole 160mg/800mg per day.

The patient was discharged on April 25, 2021, after control of PV within 2 weeks after RTX infusion. After 3 weeks, the patient failed to attend the appointment, and after contacting the family, we were informed that she passed away in another hospital, 5 days after the onset of fever, cough, and dyspnea that progressed to respiratory failure. COVID-19 was highly suspected, as the patient had close contact with a sibling with similar symptoms.

### Patient 4

A 61-year-old diabetic patient with mucocutaneous PV, with erosions on the trunk and oral mucosa since 2018, initially treated with prednisone 100 mg/day and MMF 3 g/d, progressively healed, allowing tapering of MMF from May until November 2020; by then, prednisone 15 mg/day was maintained as monotherapy due to PV remission and to a scheduled a cataract surgery.

**Figure 2:**
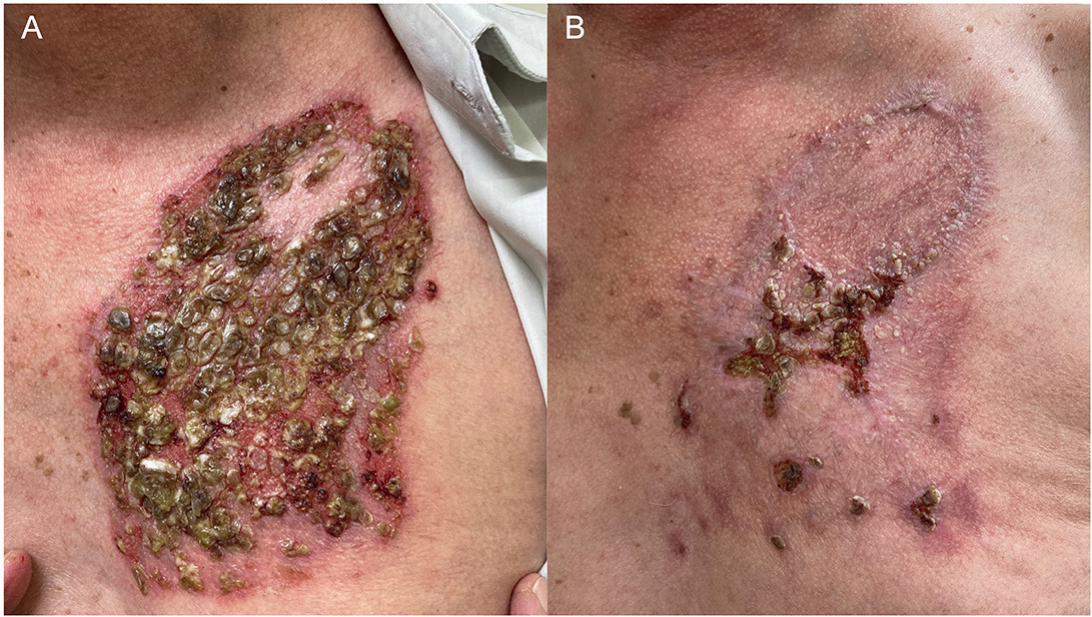
A 61-year-old man with pemphigus vulgaris had a recurrence of the disease after withdrawn of mycophenolate mofetil in November 2021 and presented **(A)** eroded plaques with purulent crusts and keratotic areas on the trunk in July 2021. Lesions were recalcitrant to prednisone 1 mg/kg/d and mycophenolate mofetil 3 g/d, and only improved 1 month after 2 rituximab infusions **(B)**.

In March 2021, he developed blisters and erosions on the oral mucosa, malar region and trunk that did not improve even after reintroduction of MMF 3 g/d and increase in prednisone to 80 mg/d (1 mg/kg/d). Secondary bacterial infection required prolonged treatment with trimethoprim/sulfamethoxazole 160/800 mg BID (Figure 2A). Despite risk factors for severe SARS-CoV-2 infection (male sex, age, diabetes), RTX therapy was scheduled 4 weeks after completion of COVID-19 vaccination. At the second RTX infusion, he developed herpes zoster successfully treated with valacyclovir 1 g 8/8 h for 14 days. PV lesions started improving right after RTX infusion, allowing tapering of prednisone and MMF. As of September 2021, the Brazilian Ministry of Health approved an additional booster COVID-19 vaccine dose for immunosuppressed patients, 4 weeks after vaccination completion. The patient received the additional COVID immunization, had no adverse effects and currently presents partial PV control on therapy Prednisone (30 mg/d) after 9 weeks of rituximab therapy (Figure 2B).

## Discussion

Brazil has been one of the epicenters of COVID-19 pandemic. Patient 1 highlights the difficult decision of treating a severe disease with immunosupressants such as systemic corticosteroids and MMF in a scenario during the beginning of the pandemic, when scientific knowledge regarding SARS-CoV-2 infection and treatment was scarce, whilst viral transmissibility was increasing (Reff >1) (4), with no perspective on COVID-19 immunization. He presented a severe, refractory bullous-invasive PF, only controlled with the association of prednisone and MMF.

MMF is a first line adjuvant drug in the treatment of AIBD (5) due to its corticosteroid-sparing effect with a better safety profile, when compared to other immunossupressants. MMF selectively inhibits *de novo* purine synthesis of B- and T-cells, and its active metabolite—mycophenolic acid—presents a half-life of 17.9 h (6).

Low lymphocyte levels are considered predictors of poor outcome in COVID-19 (7). The use of MMF during the pandemic became a great concern, once lymphopenia is a potential adverse effect of the drug (6). In COVID-19, it has been hypothesized that SARS-CoV-2 may present direct cytotoxic effects in lymphocytes, as they also express ACE2 receptor, or that lymphopenia may be a result of a dysregulated immune response to the virus and to the corticosteroid treatment for the infection (7). On the other hand, *in vitro* studies demonstrated an antiviral effect of mycophenolic acid at a concentration of 0.87 μm/mL (8), which is much lower than the therapeutic level of 1.2–8.0 μm/mL observed in patients during MMF treatment of 1–3.5 g/d (9).

A systematic review including eight studies with 732 patients with AIBD under immunomodulatory (corticosteroid, MMF, azathioprine, RTX) treatment observed no increased risk of severe SARS-CoV-2 or mortality in comparison with the general population (10). However, heterogeneity in the studied population including different AIBD with variable disease activity and treatment regimens requires caution to interpret the data.

A committee of experts currently recommends to withdraw MMF treatment during active COVID-19 (11). For patients with adequate AIBD control, it is advisable to outweigh benefits and risks of maintenance therapy with MMF. Current studies suggest mortality rates among patients with bullous pemphigoid are higher than age-matched controls (12). As potentially life-threatening diseases, AIBD flares may also require higher doses of systemic corticosteroid and hospitalization, thus aggravating the infectious risk. A retrospective single-center study demonstrates that prednisone >10 mg/d increases the risk of COVID-19 hospitalization and mortality (13).

Patient 2 had COVID-19 after 2 months of RTX treatment. He had additional risk factors for poor outcome including gender (male), obesity and diabetes; furthermore, vaccination was not available to him (young patient on immunosupressants). Nevertheless, full recovery was achieved due to adequate intensive care support at a reference hospital for COVID-19, and new recommendations for the management of severe pulmonary SARS-CoV-2 infection: anticoagulation (14), oxygen supplementation, dexamethasone (15), and awake proned ventilation (16).

Rituximab is an IgG anti-CD20 monoclonal antibody that promotes B-cell depletion and reduces antibody synthesis for 6–12 months (17). CD20+ cell recovery usually occurs within 6 to 9 months after the infusion (18). Though this prolonged effect enables AIBD remission with lower cumulative corticosteroid dose, it poses a challenge during the COVID-19 pandemic, as patients may experience a higher infectious risk and disease severity. Current studies demonstrated that AIBD and rheumatic patients treated with rituximab have an increased risk of COVID-19 mortality that reduces monthly after the infusion following B-cell recovery (11).

A retrospective study analyzed the outcomes of COVID-19 in 19 AIBD patients with confirmed SARS-CoV-2 infection. Among patients with bullous pemphigoid (*n* = 11), pemphigus vulgaris (*n* = 4), pemphigus foliaceus (*n* = 3) and mucous membrane pemphigoid (*n* = 1), the only 2 deaths occurred in patients who had been treated with rituximabe <6 months before COVID-19: a 74-year-old male PV patient with hypertension that received rituximab 2 months before the infection and a 82-year-old female BP patient with hypertension, dementia and chronic obstructive lung disease that was treated with rituximab 4 months prior to SARS-CoV-2 infection (19). Another retrospective cohort study evaluated COVID-19 outcomes in 704 AIBD patients and observed that a decrease of 38% in the relative risk of SARS-CoV-2 infection and of 45% in the relative risk of hospitalization occurs every month after rituximab infusion (13). This suggests that B-cell depletion increases the COVID-19 severity (19). As humoral response recovery is crucial for adequate response to vaccination against SARS-CoV-2, it is currently recommended to postpone rituximab infusion at least 4 weeks after vaccination completion (11).

An observational study including 3,729 patients with rheumatic diseases and suspected or confirmed COVID-19 diagnosis demonstrated that patients treated with rituximab have a 4.04 increased risk of mortality in comparison to patients receiving methotrexate in monotherapy. Limitations included a potential reporting bias, as this physician-registry study may have included more severe cases, and missing data concerning the interval between last rituximab infusion and SARS-CoV-2 infection (20).

For these reasons, maintenance treatment with rituximab infusions in patients under disease control has been discouraged. Updated expert opinion recommends treatment with rituximab for patients with recalcitrant disease and without comorbidities (17).

Patient 3 also developed COVID-19 during an active phase of anti-CD20 treatment. She received RTX due to recalcitrant PV despite high dose prednisone and MMF, that led to a prolonged hospitalization. Immunosuppressive therapy with corticosteroid and RTX, lymphopenia and bilateral pulmonary embolism may have contributed to a poor outcome despite anticoagulation therapy with rivaroxaban. Unfortunately, even after extensive evaluation, the cause of her pulmonary embolism remained undetermined and may have been related to PV activity.

Previous studies revealed that patients with active pemphigus and bullous pemphigoid have higher risk of venous thromboembolism, possibly related to increased expression of tissue factor and pro-inflammatory cytokines leading to a prothrombotic state (21, 22). An Italian multicenter cohort study demonstrated a 15-fold risk of venous thromboembolism in patients with active BP (21), whereas a Israeli population-based study showed a 2-fold risk of pulmonary embolism in pemphigus patients, mainly during the first year of the disease (22). Additional studies are necessary to determine the benefits of thromboprophylaxis in such patients, especially in the context of COVID-19 pandemic, as the SARS-CoV-2 infection may further activate the coagulation cascade and increase the risk of life-threatening thromboembolic events (14, 23).

Patient 4 presented reactivation of PV lesions following MMF withdrawn without improvement, with reintroduction of MMF treatment and increase in prednisone daily dose. He received extensive explanations regarding potential risks and benefits of rituximab therapy, as well as safety measures to prevent COVID-19 infection. Considering current knowledge regarding the outcomes of SARS-CoV-2 infection in patients with AIBD, the availability of COVID-19 vaccine enabling a reduction in the number of new cases and viral transmissibility in Brazil, we scheduled rituximab infusions in September 2021, at a better pandemic scenario than patients 2 and 3 and 4 weeks after vaccination completion.

Randomized controlled trials focusing on the approval of COVID-19 vaccines demonstrated efficacy and safety only among healthy individuals and did not include immunosuppressed patients with autoimmune diseases. Pre-pandemic studies demonstrated that the vaccine response may also be impaired in patients treated with RTX. A systematic review and meta-analysis of 38 studies including 905 patients with autoimmune disorders or hematologic malignancies evaluated the immune response of RTX-treated patients to different vaccines. A lower vaccine response in patients treated with RTX was observed in comparison with disease controls treated with other immunosuppressants and healthy individuals, with seroconversion rates from 0 to 25% in patients under active treatment (<12 weeks between RTX infusion and vaccination) (24).

Immune responses to novel technologies incorporated in SARS-CoV-2 vaccines, such as lipid-nanoparticles including mRNA of S1 receptor binding domain used in BNT162b2 (Pfizer-BioNTech) and mRNA-1273 (Moderna), are being further evaluated in AIBD patients. It has been hypothesized that upregulation of interferon-I following COVID-19 vaccination may induce autoimmunity and trigger the onset of AIBD or disease relapse (25). Moreover, immune dysregulation induced by vaccination may precipitate an epitope spreading phenomenon thus leading to recognition of self-antigens (26), and clonal expansion of T cells exhibiting SARS-CoV-2 reactivity (27). Lesion development has been reported between 1 day and 3 weeks after the first and/or second vaccination. Current data supports vaccination completion even for patients that experienced disease flares after the first dose, as seroconversion has been documented and adequate AIBD control may be achieved with appropriate treatment adjustment (28). Observational studies including patients with immune-mediated inflammatory diseases demonstrated a similar adverse effect and safety profile as in healthy individuals (25). It is noteworthy that additional studies to evaluate COVID-19 outcomes in vaccinated AIBD patients are necessary to better understand the safety of immunosuppressive and immunobiologic treatments after immunization.

From March 2020 on, management of AIBD during pandemic is evolving along with advances in vaccination and COVID-19 treatment, although an effective and specific antiviral therapy is still missing. As a reference center for AIBD patients, we are currently receiving patients with uncontrolled disease because of initial pandemic restrictions limiting access to health care facilities. Patients are encouraged to receive COVID-19 vaccination including the booster dose, and to maintain protective measures to prevent SARS-CoV-2 infection (social distancing and protective personal equipment). For severe AIBD cases, RTX treatment is scheduled at least 4 weeks after full COVID-19 vaccination. We are now considering postponing RTX infusions following the novel recommendation to perform a booster dose at least 4 weeks after full vaccination completion. After anti-CD20 therapy, B-cell recovery monitoring may help to determine the most appropriate timing to vaccine patients to maximized seroconversion. A recent study demonstrated that CD19+ recovery is a predictor of adequate immune response after vaccination (29). In accordance to Shakshouk et al. (30), we are performing SARS-CoV-2 PCR for screening before each infusion. Meanwhile, outpatient evaluations are scheduled in a way to minimize hospital visits while maintaining frequent monitoring to adjust corticosteroid and immunosuppressants dosage to the lowest possible.

## Data Availability Statement

The original contributions presented in the study are included in the article/supplementary material, further inquiries can be directed to the corresponding author/s.

## Ethics Statement

This study was reviewed and approved by the Ethics Committee of our institution (Comissão de Ética para Análise de Projetos de Pesquisa - CAPPesq # 56796222.6.0000.0068). All patients or next of kin provided written informed consent to participate in this manuscript and for the publication of any potentially identifiable images or data included in this article.

## Author Contributions

All authors listed have made a substantial, direct, and intellectual contribution to the work and approved it for publication.

## Conflict of Interest

The authors declare that the research was conducted in the absence of any commercial or financial relationships that could be construed as a potential conflict of interest.

## Publisher's Note

All claims expressed in this article are solely those of the authors and do not necessarily represent those of their affiliated organizations, or those of the publisher, the editors and the reviewers. Any product that may be evaluated in this article, or claim that may be made by its manufacturer, is not guaranteed or endorsed by the publisher.
